# Unexpected cause of recurrent diabetic ketoacidosis in type 1 diabetes: a case report

**DOI:** 10.1186/s12902-023-01394-3

**Published:** 2023-07-03

**Authors:** Rabia Khalid Alduraibi, Yosef Fahad Altowayan, Bader Tha’ar AlMharwal

**Affiliations:** 1 Department of Endocrine and Diabetes, King Fahad Specialist Hospital, Box 3499, Buraydah, 52385 – 669 , Saudi Arabia; 2 Department of Internal Medicine, King Fahad Specialist Hospital, Buraydah, Saudi Arabia

**Keywords:** Diabetic ketoacidosis, Cannabinoid hyperemesis syndrome, Type 1 diabetes, Cannabis

## Abstract

**Background:**

Gastrointestinal (GI) symptoms are commonly observed in patients with diabetic ketoacidosis (DKA), which usually resolves completely with therapy. However, GI symptoms can persist after DKA resolves, which can pose diagnostic and management challenges for physicians, especially when dealing with an exceptional diagnosis such as cannabinoid hyperemesis syndrome (CHS).

**Case presentation:**

In this case report, we present a patient with type 1 diabetes who had been treated for DKA 6 times in the past year and was eventually diagnosed with CHS.

**Conclusion:**

In conclusion, this case demonstrates that a presumptive and incorrect diagnosis can mislead physicians, especially when dealing with challenging diagnoses. Therefore, patients with type 1 diabetes with unusual presentations, such as unexpectedly high pH and bicarbonate levels, with hyperglycemic ketosis should be screened for illicit drug use, especially cannabis.

## Background

Diabetic ketoacidosis (DKA) is a serious life-threatening condition that usually requires admission to the intensive care unit (ICU). The development of DKA has been associated with a number of precipitating factors such as inadequate insulin therapy, infection, ischemia, medications, and other medical-surgical conditions. The absence of clear precipitating factors may make DKA diagnosis and treatment challenging and can easily lead to misdiagnosis.

Cannabis use should be considered in patients with type 1 diabetes who have recurrent DKA with an unusual presentation, such as unexpectedly high pH and bicarbonate levels with hyperglycemic ketosis.

Here, we describe a patient with type 1 diabetes who presented with recurrent DKA, abdominal pain, and cyclic vomiting thought to be due to cyclic vomiting syndrome. After an appropriate evaluation, the patient was diagnosed with cannabinoid hyperemesis syndrome (CHS (. This case provides an opportunity to discuss the diagnosis and treatment of this challenging condition.

As far as we know, this is the first case report on this topic in Saudi Arabia.

### Case presentation

A 21-year-old women was diagnosed with type 1 diabetes at the age of 7 years. She also had anxiety and depression that required expert help. Basal bolus insulin was used to treat her diabetes. She had a history of frequent admissions for DKA 6 times within the last 12 months and a presumptive diagnosis of cyclic vomiting syndrome. All DKA episodes started with sudden onset of nausea, vomiting, and abdominal pain that worsened over time, eventually leading to dehydration and ketosis. DKA episodes were managed according to the standard protocol. There was no apparent cause for any of the episodes of DKA, either in her history or in extensive investigations. On the last admission, she presented with abdominal pain, nausea, and vomiting. She reported 7 episodes of bilious vomiting one day prior to admission. The pain was epigastric, 7/10 in intensity, progressive, and nonradiating. The pain was mainly postprandial and accompanied by vomiting, which was resolved with paracetamol. She denied any aggravating factors, but the hot water baths provided some relief.

Physical examination revealed a mild epigastric. The initial laboratory test revealed DKA. Her arterial pH was 7.27 (normal value: 7.32–7.43), her serum bicarbonate level was 12.7 mmol/l (normal value: 22–29 mEq/L), her pCO2 was 21.3 mmHg (normal value: 38–42 mmHg), her anion gap was 22 (normal value: 3–10 mEq/L), and her blood glucose level was 20.3 mmol/l (normal value: 3.8–5.5 mmol/l). Her HbA1c was 7.9% (normal value: 4–6.5%). The white blood cell count was 11.3/μL (normal value: 4–11.0 × 10^9^/L), haemoglobin was 11.4 g/d (normal value: 12–16 g/d), sodium was 134 mEq/L (normal value: 135–145 mEq/L), potassium was 4.2 mEq/L (normal value: 3.5–5.5 mEq/L), chloride was 108 mEq/L (normal value: 96–106 mEq/L), aspartate aminotransferase was 54 U/L (normal value: 8–40 U/L), alanine aminotransferase was 27 U/L (normal value: 5–56 U/L), total bilirubin was 4.7 µmol/L (normal value: 3.4–20.5 µmol/L), and amylase was 32 U/L (normal value: 28–100 U/L). The dipstick urine test revealed the present of 2 + ketones and 4 + glucose. Enhanced abdominal and pelvis computed tomography (CT) was performed to rule out celiac artery compression syndrome (CACS), which was unremarkable. Furthermore, she underwent an abdominal ultrasound, and upper GI endoscopy was normal. Toxicology screening revealed positivity for urinary cannabinoids. The patient was a regular cannabis user with no history of other recreational drug use. After taking a thorough history, it was found that her vomiting had been triggered by more cannabis use than usual.

Treatment with intravenous fluid and insulin was initiated immediately. Her DKA responded within 24 h of this medical management. CHS was diagnosed after a comprehensive review of the patient's medical history, physical examination, and a strong correlation between cyclic vomiting and cannabis use. Other etiologies, such as gastroparesis and CACS were ruled out with additional testing. The patient was informed of the diagnosis and advised to stop cannabis use.

## Discussion

Chronic gastrointestinal (GI) symptoms, including vomiting and abdominal pain, are most commonly observed among patients with type 1 diabetes, for which presumptive and incorrect diagnoses can mislead physicians, especially when dealing with rare diagnoses of exclusion, such as CHS.

To our knowledge, this is the first case report of an association between HCS and recurrent DKA in patients with type 1 diabetes in Saudi Arabia. This can be challenging to diagnose due to a variety of factors.

Cannabis is the most commonly used psychoactive drug in the world. According to the latest World Drug Report 2019 of the United Nations (UNODC), an estimated 271 million people (5.5% of the world population aged 15–64 years) had used drugs at least once in the previous year [1].

The use of cannabis by Saudis with type 1 diabetes has not been well described in the literature. Few published studies have estimated that approximately 7–8% of Saudis have used drugs [2, 3]. The substances most frequently abused by Saudis are amphetamines, heroin, alcohol, and cannabis [4].

Patients with type 1 diabetes who use cannabis are more likely to experience recurrent vomiting, and abdominal pain, which can be difficult to diagnose and manage. Similar symptoms may be experienced by those with diabetic gastroparesis. Therefore, a comprehensive, detailed medical history and physical exam are important in identifying cause and avoiding unnecessary tests and procedures.

Long-term regular cannabis use can cause CHS, a rare condition characterized by nausea progressing to severe vomiting that occurs in cycles leading to dehydration and ketosis, followed by hyperglycaemia [5, 6]. In contrast to typical DKA, in which ketoacidosis is preceded by hyperglycemia mainly due to insulin omission or other precipitating factors. Cannabis use may mask DKA by causing an increase in pH documented at the time of presentation compared to non-cannabis users with DKA. The mechanism for this high pH is related to the effect of cannabis on delaying gastric emptying and progressive emesis, leading to metabolic alkalosis [7]. CHS can present as recurrent DKA, as in this case [8].

Before settling on a diagnosis of CHS, it’s important to exclude other critical conditions based on risk factors, some of which may necessitate emergency surgery and treatment. Therefore, it is necessary to conduct an appropriate workup, which should include a complete blood count, a metabolic panel to evaluate electrolyte disturbance and the degree of dehydration. Imaging is performed at the discretion of the clinician based on a variety of aspects, including medical history and a physical examination. In patients with type 1 diabetes gastroscopy and gastric emptying studies are impotent to rule out diabetic gastroparesis.

Several checklists have been developed to help diagnose CHS, the most recent being the Rome IV criteria. A review of the literature reveals that the diagnosis of CHS has been characterized by diagnostic delays and an increased number of visits to the emergency department prior to diagnosis [9]. Similarly, our patient has a history of chronic cannabis use, multiple admissions with episodes of abdominal pain, and cyclic vomiting that is partially relieved by hot water baths. She underwent a comprehensive examination and workup that was negative for any underlying pathology.

Stopping cannabis use is the main treatment option for CHS. Hot baths may temporarily relieve nausea but do not treat CHS. In the acute setting, supportive therapy with intravenous fluids and anti-emetics remains the mainstay of treatment. The use of benzodiazepines, haloperidol, and capsaicin has been suggested to treat the acute phase of CHS [10]. Unfortunately, there are currently no medications that have been shown to be effective in treating cannabis withdrawal. Short-term symptomatic drugs may be helpful. For our patient, it was difficult to prevent repeat hospitalizations because of an ongoing problem with cannabis use.

## Conclusion

In conclusion, with the increasing use of cannabis and cannabis-related emergency visits among adults with type 1 diabetes, physicians should differentiate between typical DKA and CHS. This can prevent patients from having to undergoing unnecessary invasive tests that expose them to radiation.

## Data Availability

The data generated during this study were included in this published article.

## Abbreviations

GI: Gastrointestinal
CHS: Cannabis hyperemesis syndrome
DKA: Diabetic Ketoacidosis
CT: Computed tomography
ICU: Intensive care unit
UNODC: United nations office on drugs and crime
CACS: Celiac artery compression syndrome
